# Predicting response and toxicity to immune checkpoint inhibitors using routinely available blood and clinical markers

**DOI:** 10.1038/bjc.2017.274

**Published:** 2017-08-24

**Authors:** Ashley M Hopkins, Andrew Rowland, Ganessan Kichenadasse, Michael D Wiese, Howard Gurney, Ross A McKinnon, Chris S Karapetis, Michael J Sorich

**Affiliations:** 1 Flinders Centre for Innovation in Cancer, College of Medicine and Public Health, Flinders University, Flinders Drive, Bedford Park, Adelaide, South Australia 5042, Australia; 2 Department of Clinical Pharmacology, College of Medicine and Public Health, Flinders University, Flinders Drive, Bedford Park, Adelaide, South Australia 5042, Australia; 3 School of Pharmacy and Medical Sciences, University of South Australia, Frome Street, Adelaide, South Australia 5000, Australia; 4 Department of Medical Oncology, Westmead Hospital, Hawkesbury Road & Darcy Road, Westmead, Sydney, New South Wales 2145, Australia

**Keywords:** immune checkpoint inhibitors, precision medicine, biomarkers, clinicopathological, melanoma

## Abstract

Immune checkpoint inhibitors (ICI) are an important development in the treatment of advanced cancer. A substantial proportion of patients treated with ICI do not respond, and additionally patients discontinue treatment due to adverse effects. While many novel biological markers related to the specific mechanisms of ICI actions have been investigated, there has also been considerable research to identify routinely available blood and clinical markers that may predict response to ICI therapy. If validated, these markers have the advantage of being easily integrated into clinical use for nominal expense. Several markers have shown promise, including baseline and post-treatment changes in leucocyte counts, lactate dehydrogenase and C-reactive protein. While promising, the results between studies have been inconsistent due to small sample sizes, follow-up time and variability in the assessed markers. To date, research on routinely available blood and clinical markers has focussed primarily on ICI use in melanoma, the use of ipilimumab and on univariate associations, but preliminary evidence is emerging for other cancer types, other ICIs and for combining markers in multivariable clinical prediction models.

Immune checkpoint inhibitors (ICIs), particularly inhibitors of cytotoxic T-lymphocyte antigen-4 (CTLA-4), programmed death receptor-1 (PD-1) and its associated ligand (PD-L1), represent an important development in the treatment of advanced cancers (Champiat *et al*, 2016). Unfortunately a substantial proportion of patients treated with ICIs do not respond, while a small proportion of those with survival benefit display a period of apparent treatment failure (pseudoprogression) at the commencement of therapy (Henze *et al*, 2016). Additionally, ICI use is associated with a spectrum of unique and potentially severe toxicities termed immune-related adverse events (irAEs) (Champiat *et al*, 2016). Patients may discontinue treatment due to irAEs in a setting, where the necessary duration of treatment is unclear.

Immune checkpoint inhibitors appear capable of producing durable responses compared to existing treatments in a subset of patients with advanced melanoma. Ipilimumab is an anti-CTLA-4 monoclonal antibody (mAb), and, although the proportion of melanoma patients who appear to benefit from treatment remains modest, there is approximately a 10% increase (doubling) of the survival at 5 years compared to cytotoxic chemotherapy (Garbe *et al*, 2016; Maio *et al*, 2015). Additionally, there was a very low mortality rate observed between 3 and 5 years of follow-up (Maio *et al*, 2015), providing hope that these individuals may continue to respond for many more years. The PD-1 inhibitors, nivolumab and pembrolizumab, are able to achieve a response in a larger proportion of melanoma patients, and although long-term survival data on these therapies are not yet mature, preliminary results are promising (Postow *et al*, 2015; Ribas *et al*, 2015; Robert *et al*, 2015; Robert *et al*, 2015; Weber *et al*, 2015; Seetharamu *et al*, 2016; Topalian *et al*, 2016). Combination therapy with ipilimumab and a PD-1 inhibitor may further improve response and survival in advanced melanoma, but greater rates of toxicity may occur compared to PD-1 therapy alone (Postow *et al*, 2015; Topalian *et al*, 2016).

Substantial survival benefits with nivolumab and pembrolizumab have also been demonstrated for other cancers, including non-small-cell lung cancer (NSCLC), urothelial cancer, metastatic renal cell carcinoma (mRCC) (Borghaei *et al*, 2015; Brahmer *et al*, 2015; Garon *et al*, 2015; Motzer *et al*, 2015; Champiat *et al*, 2016; Bellmunt *et al*, 2017; Sharma *et al*, 2017). Preliminary outcomes from trials evaluating other non-approved anti-PD-1 (pidilizumab) and anti-PD-L1 (atezolizumab, durvalumab, avelumab and BMS-936559) mAbs are also displaying promising response rates for a range of cancers (Barbee *et al*, 2015; Rittmeyer *et al*, 2016; Rosenberg *et al*, 2016; Seetharamu *et al*, 2016). If these ongoing trials confirm the expected effects on survival, there will be significant growth in the patient populations using ICIs, and thus optimising outcomes becomes increasingly important.

There has been extensive research of novel biological markers that are specific to the mechanism of actions of ICI that may predict response to therapy and these markers have been recently and extensively reviewed (Meng *et al*, 2015; Topalian *et al*, 2016; Gnjatic *et al*, 2017). In parallel, there has also been considerable research conducted to identify if any routinely available blood and clinical markers are predictive of response and toxicity to ICIs. If validated to be predictive, routinely available blood and clinical markers have the advantage of being readily available in the clinic, and hence easily and quickly integrated in clinical decision-making. It is biologically plausible that some routinely available markers, such as peripheral blood lymphocyte count, may provide insight into the activity of the immune system and hence provide the capacity for the immune system to mediate a strong antitumour effect in the presence of ICI therapy (Pardoll, 2012). The association between routinely available blood and clinical markers and ICI response/toxicity is, therefore, the focus of this review.

## Search process

Studies investigating the association between routinely available blood and clinical markers and ICI response/toxicity were identified through a structured search of Scopus and then Google Scholar in July 2017. The search terms included the name of FDA approved ICI’s (atezolizumab, avelumab, durvalumab, ipilimumab, nivolumab and pembrolizumab), ‘biomarker’ OR ‘marker’ OR ‘predictor’, plus ‘response’ OR ‘survival’ OR ‘toxicity’. Studies were included if they reported investigation of the association between routinely available blood and clinical markers and ICI response or toxicity. References and citations of selected studies were hand-searched for reference to any additional relevant studies.

## Potential predictors of ICI efficacy

The relatively modest response rate with ICI therapy, coupled with the potential to achieve long-term response in those who do respond, suggests that the discovery of markers that predict ICI efficacy would be useful. Many biomarkers are being explored for ICI therapy and these are reviewed in depth elsewhere (Meng *et al*, 2015; Topalian *et al*, 2016; Gnjatic *et al*, 2017). In brief, predictive biomarkers proposed for ipilimumab response include baseline expression of CD4^+^ICOS^high^ and Ki67^+^EOMES^+^CD8^+^ T-cells, increased FOXP3 and indoleamine 2,3-dioxygenase expression, and reduced expression of regulatory T cells (Ascierto *et al*, 2013). Circulating baseline levels of TGF-*β*1 and IL-10 are also proposed prognostic markers for relapse following ipilimumab therapy. Expression of PD-L1, particularly on infiltrating myeloid and T cells, but not tumour cells, is currently a promising predictive biomarker of response for anti-PD-1/PD-L1 mAbs, and positive expression of PD-L1 is associated with improved response rate, progression-free survival and overall response in a number of studies (Meng *et al*, 2015; Topalian *et al*, 2016). However, PD-L1-negative tumours may still respond to treatment. While mechanistically plausible, there is currently limited evidence for genetic and epigenetic markers such as miR34 expression (Remon *et al*, 2016). Exploratory analyses have shown The Cancer Genome Atlas (TCGA) subtypes and mutation load to be predictive of response to atezolizumab used in the treatment of metastatic urothelial carcinoma (Rosenberg *et al*, 2016). Programmed death receptor ligand-2, interferon gamma, *EGFR* mutations and anaplastic lymphoma kinase (ALK) rearrangements may represent novel biomarkers that could be explored further in the future (Gainor *et al*, 2016; Remon *et al*, 2016).

While the above-mentioned biomarkers may predict efficacy and improved response rates to ICIs, there would be a cost to integrating their measurement into clinical care. In contrast, several small retrospective investigations have evaluated routinely available blood and clinical markers that may predict therapeutic benefit from ICIs (Table 1). To date, the majority of investigations have focussed on ipiliumumab, nivolumab or pembrolizumab in the treatment of melanoma. Baseline and post-treatment changes in leucocyte counts, lactate dehydrogenase (LDH) and C-reactive protein all show promise as predictive biomarkers for response (Table 1). A recent report highlights that smoking status may also be relevant (Hellmann *et al*, 2014), while the pattern of visceral metastasis has also been associated with changes to survival outcomes (Weide *et al*, 2016). Adverse events may also be a possible determinate of response to ICI therapy, albeit reports are inconsistent at this stage (Table 1).

### Leucocyte count

Baseline and post-treatment changes in leucocytes including lymphocytes, eosinophils, neutrophils, neutrophil to lymphocyte ratio and monocytes counts are promising routinely available blood markers that have shown associations with response to ICI therapy (Table 1). Baseline changes in myeloid-derived suppressor cells (MDSCs) (Martens *et al*, 2016a) and regulatory T cells (Martens *et al*, 2016a, 2016b) have also been associated with response to ICI therapy but are not currently routinely available leucocyte markers. Several of these leucocyte markers have shown associations across multiple studies with the direction of response generally aligning. However, differences between study designs, methodology, marker measurement and marker use have limited the ability to identify the effect size. In particular, there are significant inconsistencies between the leucocytes measured, the use of absolute or relative counts, the use of a baseline or a landmark analysis approach and the marker cut-point that most clearly distinguishes individuals likely and unlikely to respond to therapy.

As ipilimumab blocks CTLA-4 expressed on various lymphocyte populations, a high peripheral blood lymphocyte count may reflect a greater capacity of the immune system to mediate a strong antitumour effects in the presence of ipilimumab (Ku *et al*, 2010). Accordingly, the potential association between lymphocyte counts and ipilimumab response has been investigated in several studies. In melanoma patients treated with ipilimumab, high and increased absolute lymphocyte counts (ALC) at 2–12 weeks after treatment initiation have been associated with improved response and overall survival (OS) (Delyon *et al*, 2013; Ku *et al*, 2010; Martens *et al*, 2016b; Simeone *et al*, 2014). These results have been demonstrated in small cohorts ranging from 51 to 95 melanoma patients treated with ipilumumab at 3 and 10 mg kg^−1^ every 3 weeks at European and American sites (Delyon *et al*, 2013; Ku *et al*, 2010; Martens *et al*, 2016b; Simeone *et al*, 2014). Martens *et al* (2016a) did not confirm these results, but did find that an increased relative lymphocyte count (RLC; percent of leucocytes that are lymphocytes) at baseline was associated with improved OS (*n*=204). In one of the largest studies to investigate an association between lymphocytes and response to ICI to date (*n*=616, European and American melanoma patients), no association was found with ALC, but increased RLC at baseline was associated with improved OS (Weide *et al*, 2016). Similarly, Wolchok *et al* (2013) found no association between increased ALC and response in melanoma patients treated with nivolumab and ipilimumab, although the study population was small (*n*=53) and did not assess RLC. Similar inconsistencies in results have been demonstrated for eosinophil and neutrophil counts, and for neutrophil to lymphocyte ratios (Delyon *et al*, 2013; Wolchok *et al*, 2013; Ferrucci *et al*, 2015; Gebhardt *et al*, 2015; Ferrucci *et al*, 2016; Martens *et al*, 2016a; Weide *et al*, 2016; Zaragoza *et al*, 2016).

Despite these inconsistencies, leucocytes counts are among the most promising routinely available blood markers that may be able to predict response to ICI therapy. For example, Ku *et al* (2010) indicated that an ALC>1000 cells per *μ*l at week 7 correlated with a significantly improved clinical benefit rate (17 of 33 patients (51%) *vs* 0 of 8; *P*<0.01) and median OS (11.9 *vs* 1.4 months; *P*<0.001) compared with those with an ALC<1000 cells per *μ*l. While Ferrucci *et al* (2016) indicated that patients with an absolute neutrophil count (ANC)>7500 cells per *μ*l and a derived neutrophil/lymphocyte ratio (dNLR)>3 had a significantly increased risk of death (hazard ratio (HR)=5.76; 95% confidence interval (CI) 4.29–7.75) and disease progression (HR=4.10; 95% CI 3.08–5.46) compared to patients with a lower ANC and dNLR. Such results indicate that leucocyte and leucocyte sub-type counts may be able to be used in the clinic to spare patients potentially ineffective or toxic treatments, and thus allow the commencement of alternate treatments.

Variability in study design makes it difficult to compare results across studies. For example, Ferrucci *et al* (2016) conducted the largest study to date to assess leucocytes associations with response to ipilimumab treatment in melanoma patients (*n*=720, Italian melanoma patients treated with 3 mg kg^−1^ of ipilimumab every 3 weeks). However only absolute neutrophil and total leucocyte counts were available to researchers, but not lymphocyte, monocyte, eosinophil and basophil counts. Thus, it would be desirable to conduct a large study assessing all the routinely collected leucocyte counts to determine the most suitable marker of response/toxicity.

### Lactate dehydrogenase

Elevated LDH levels are a prognostic factor for poor survival outcomes in patients with metastatic melanoma, mRCC and many other tumour types. This is recognised by the American Joint Committee on Cancer (AJCC), which includes LDH levels as part of their melanoma staging and classification system (Balch *et al*, 2009). Normal baseline LDH is associated with improved response and OS in melanoma patients treated with ipilimumab, pembrolizumab and nivolumab (Delyon *et al*, 2013; Simeone *et al*, 2014; Valpione *et al*, 2015; Collins and Le Manach, 2016; Diem *et al*, 2016; Khoja *et al*, 2016; Weide *et al*, 2016; Zaragoza *et al*, 2016; Martens *et al*, 2016a). The potential clinical importance of this finding is reflected in a real-world cohort of melanoma patients treated with nivolumab or pembrolizumab, in which half had elevated LDH levels at baseline (Diem *et al*, 2016). Post treatment increases in LDH levels were also associated with poorer response and survival in this cohort (Diem *et al*, 2016). Further demonstrating the potential clinical importance of LDH levels is the multivariable analysis conducted by Martens *et al* (2016a), which identified that normal baseline LDH, absolute monocyte counts, MDSCs frequencies, absolute eosinophil count, RLC and regulatory T cells (Treg) frequencies were associated with improved survival in ipilimumab-treated melanoma patients. In this analysis, LDH was a strong predictor of improved outcomes, with a median OS of 10 months for patients with baseline LDH up to 1.2-fold higher than the upper limit of normal, while for those >1.2- and >2.3-fold, it was only 5 and 2 months, respectively (*P*<0.0001) (Martens *et al*, 2016a).

### Adverse events

Adverse events have been associated with response to a number of cancer medicines, in particular the targeted medications. For example, proteinuria was recently identified as being associated with improved survival in mRCC patients treated with vascular endothelial growth factor targeted agents (Sorich *et al*, 2016). In a meta-analysis of 137 studies evaluating cancer immunotherapies (including 11 general immune stimulation, 84 vaccine, 28 antibody-based and 16 adoptive transfer treatment arms), a strong association between vitiligo-like depigmentation and survival was also identified (*P*<0.024), but the association for ICI therapies specifically is unknown (Teulings *et al*, 2015). Since that time the irAE vitiligo has also been associated with improved objective response in a melanoma cohort treated with pembrolizumab (Hua *et al*, 2016), and survival in a melanoma cohort treated with nivolumab (Freeman-Keller *et al*, 2016). However both studies were relatively small and evidence on whether irAE are predictive of ICI response/survival, including but not limited to vitiligo, requires clarification in larger studies (Weber *et al*, 2017). Greater exposure to ipilimumab (i.e., higher plasma drug concentrations) is associated with increased response/survival and higher rates of irAEs (Feng *et al*, 2013), which is suggestive that irAE may predict response and survival.

## Potential predictors of ICI toxicity

Immune checkpoint inhibitors have been associated with severe irAEs such as rash, diarrhoea, colitis, hypophysitis, hepatotoxicity and hypothyroidism (Champiat *et al*, 2016). Severe irAEs are more common with ipilimumab (15–43% of patients) than nivolumab or pembrolizumab. However, ∼10–20% of patients treated with anti-PD-1 mAbs still develop severe, potentially life-threatening toxicities, and this increases further when combining with anti-CTLA-4 and anti-PD-1 mAbs (Postow *et al*, 2015; Champiat *et al*, 2016; Topalian *et al*, 2016). Potential predictors of ICI toxicity and irAEs have been less thoroughly investigated than predictors for response. Although, the presence of baseline sarcopenia and low muscle attenuation were recently associated with the occurrence of severe treatment-related toxicity (Daly *et al*, 2017). Several other potential baseline risk factors for severe irAEs have also been proposed, including family history of autoimmune diseases, tumour infiltration and location, previous viral infections such as HIV or hepatitis and the concomitant use of medicines with known autoimmune toxicities such as antiarrhythmics, antibiotics, anticonvulsants or antipsychotics (Champiat *et al*, 2016; Manson *et al*, 2016). A small study recently indicated that ipilimumab-treated patients experiencing irAEs appear to present with a diversification of the T-cell repertoire (Fong *et al*, 2016; Oh *et al*, 2017), while increased eosinophil count has also been linked to irAEs (Schindler *et al*, 2014). Another small study found that increased circulating IL-17 levels might be associated with gastrointestinal toxicity (Tarhini *et al*, 2015); however in general the investigation of predictors of ICI toxicity requires increased research.

## Future perspective

Following ICI therapy initiation, some patients have an influx of effector cells to the tumour masses and an apparent increase in tumour size (pseudoprogression) (Henze *et al*, 2016). To improve the assessment of the effect of immunotherapeutic agents, the immune-related Response Evaluation Criteria in Solid Tumors (irRECIST) was developed (Henze *et al*, 2016), while research continues to explore novel methods to detect early response to ICI. These factors exemplify the importance of identifying predictive markers of response that may justify continued therapy in lieu of a traditional response profile. To facilitate the translation of identified predictors into clinical strategies, prospective investigations comparing standard practices against modified strategies will be required. In this manuscript, we have focussed on compiling the studies that have identified routinely available blood and clinical markers associated with response and toxicity to ICIs. The benefits of such markers are that once validated they will generally be easily available and not require additional costs or setup to integrate into clinical care. Future research will also continue to explore other biomarkers routinely collected in the clinic that may predict response to ICI therapy. Biological plausibility and pilot investigations indicate that performance status, age, concomitant therapy (particularly high-dose corticosteroids), diversity of gut microbiome, prolactin, autoimmune diseases status, human leucocyte antigen class, DNA mismatch repair complex (MMR complex), tumour characteristics (size, location of metastases) and the level of tumour infiltrating lymphocytes are potential markers that should be more thoroughly investigated in the future (Friedman and Postow, 2016; Nishijima *et al*, 2016; Seliger, 2016; Topalian *et al*, 2016; Caponnetto *et al*, 2017; Wargo *et al*, 2017; Johnson *et al*, 2017).

To date, most of the research investigating routinely available blood and clinical markers as predictors of ICI response and toxicity has focused on ipilimumab and ICIs used in the treatment of melanoma. Recent preliminary evidence is now emerging that survival of NSCLC and urothelial cancer patients treated with ICI therapy is associated with markers such as low baseline NLR, low baseline performance status, the presence of liver metastases, increasing albumin, decreasing NLR and decreasing clearance (Bagley *et al*, 2017; Powles *et al*, 2017). Despite this there is notably less research evaluating routinely available blood and clinical markers as predictors of outcomes for the newer anti-PD-1/PD-L1 drugs and other cancer types. Given the promising evidence emerging for ICIs and their growing use, this represents an important unmet area of research and care must currently be taken in generalising the predominantly melanoma studies to other cancer types. Weide *et al* (2016) did assess melanoma patients treated with pembrolizumab, and reported a combination model (based on relative eosinophil count, RLC, LDH and the absence of metastasis other than soft-tissue/lung) that could be assessed in a randomised controlled trial to determine the predictive benefit of the model on treatment decisions. Emerging evidence also indicates that combination therapy with anti-PD-1 and anti-CTLA-4 mAbs increases response rate, albeit at the expense of increased toxicity. Thus, trials examining combination therapies will likely continue into the future. This manuscript highlights the importance of trials collecting potential biomarker data that may facilitate improved responses and toxicity avoidance in those receiving combination therapies in the future.

Of the studies presented, there is considerable variability in the collected and assessed routinely available blood and clinical markers. Given the array of potential pathways and biomarkers that have been implicated in ICI efficacy and the complexity of the tumour environment and immune function, it is likely that a combination of multiple predictors will be required to effectively predict response and toxicity of ICI therapy (Martens *et al*, 2016a). Despite this, many of the studies presented herein lacked access to all the potential biomarkers. For example Ku *et al* (2010) assessed the association of ALC with OS following ipilimumab treatment using a landmark approach adjusted for baseline LDH levels (*n*=53), while Ferrucci *et al* (2016) had the largest study population to date (*n*=720), but only had access to neutrophil and total leucocyte counts. Opposing this, Martens *et al* (2016a) assessed MDSCs and Treg frequencies, LDH, monocytes, eosinophils, lymphocytes and several other clinical characteristics for associations with OS in ipilimumab treatment melanoma patients. Such a screening processes enabled the development of a multivariable model, which may improve clinical decisions over the use of a single biomarker alone. In addition to the importance of the continued investigation of potential biomarkers in multivariable analyses, dose modification strategies and therapeutic drug monitoring techniques should also be considered as mechanisms to improve response and toxicity to ICI, but have not been extensively explored.

## Conclusion

Immune checkpoint inhibitors are an emerging option in the treatment of melanoma and other advanced cancers. However, a substantial proportion of patients do not respond to ICIs, while they can be associated with a range of potentially life-threatening irAEs. Several potential predictors of ICI response and toxicity have been proposed, including routinely available blood and clinical markers. However to date these have not been extensively explored, particularly for the newer nivolumab or pembrolizumab. Several small retrospective investigations have identified association between pre- and post-treatment blood and clinical markers, and response to ipilimumab. While promising and easy to use in the clinic, these predictive markers require validation in adequately powered and well-designed multivariable analyses.

## Figures and Tables

**Table 1:** Summary of preliminary evidence of routinely available blood and clinical markers predictive of ICI outcomes

**Marker**	**ICI therapy**	**Cancer**	***N***	**Study results**	**Reference**
Lymphocyte count	Ipilimumab	Melanoma	51, 73	⩾1000 per *μ*l at week 6→↑ OS	(Delyon *et al*, 2013; Ku *et al*, 2010)
	Ipilimumab	Melanoma	82, 40	↑ At 2–8 weeks *vs* baseline→↑ response	(Bjoern *et al*, 2016; Martens *et al*, 2016b)
	Ipilimumab	Melanoma	95	↑ At week 12 *vs* baseline→↑ OS	(Simeone *et al*, 2014)
	Nivolumab	Melanoma	98	⩾1000 per *μ*l at week 3–6→↑ OS	(Nakamura *et al*, 2016)
Relative lymphocyte count	Ipilimumab	Melanoma	209	↑ Baseline→↑ OS	(Martens *et al*, 2016a)
	Pembrolizumab	Melanoma	616	↑ Baseline→↑ OS	(Weide *et al*, 2016)
Total leucocyte count	Ipilimumab	Melanoma	59	↓ Baseline→↑ response	(Gebhardt *et al*, 2015)
Eosinophil count	Ipilimumab	Melanoma	209	↑ Baseline→↑ OS	(Martens *et al*, 2016a)
	Ipilimumab	Melanoma	59	↑ At week 3 *vs* baseline→↑ response	(Gebhardt *et al*, 2015)
	Ipilimumab	Melanoma	73	↑ At week 6 *vs* baseline→↑ OS	(Delyon *et al*, 2013)
Relative eosinophil count	Pembrolizumab	Melanoma	616	↑ Baseline→↑ OS	(Weide *et al*, 2016)
Neutrophil count	Ipilimumab	Melanoma	59	↓ Baseline→↑ response	(Gebhardt *et al*, 2015)
	Ipilimumab	Melanoma	720	↓ Baseline→↑ PFS and OS	(Ferrucci *et al*, 2016)
	Nivolumab	Melanoma	98	<4000 per *μ*l at week 3–6→↑ OS	(Nakamura *et al*, 2016)
Neutrophil/lymphocyte ratio	Ipilimumab	Melanoma	58, 185	↓ Baseline→↑ OS	(Khoja *et al*, 2016; Zaragoza *et al*, 2016)
	Ipilimumab	Melanoma	187	↓ Baseline→↑ PFS and OS	(Ferrucci *et al*, 2015)
	Nivolumab	NSCLC	175	↓ Baseline→↑ OS	(Bagley *et al*, 2017)
Derived neutrophil/lymphocyte ratio	Ipilimumab	Melanoma	720	↓ Baseline→↑ PFS and OS	(Ferrucci *et al*, 2016)
Monocyte count	Ipilimumab	Melanoma	209	↓ Baseline→↑ OS	(Martens *et al*, 2016a)
Lactate dehydrogenase	Ipilimumab		209, 73, 166, 58, 113, 183	↓ Baseline→↑ OS	(Delyon *et al*, 2013; Kelderman *et al*, 2014; Valpione *et al*, 2015; Collins and Le Manach, 2016; Dick *et al*, 2016; Khoja *et al*, 2016; Zaragoza *et al*, 2016; Martens *et al*, 2016a)
	Nivolumab	Melanoma	98	↑ Baseline→↓ OS	(Nakamura *et al*, 2016)
	Pembrolizumab	Melanoma	616	↓ Baseline→↑ OS	(Weide *et al*, 2016)
	Pembrolizumab, nivolumab	Melanoma	66	↓ Baseline→↑ OS ↑ At week 12 *vs* baseline→ ↓Response, OS	(Diem *et al*, 2016)
	Ipilimumab	Melanoma	95	↓ At week 12→↑ response and OS	(Simeone *et al*, 2014)
C-reactive protein	Ipilimumab	Melanoma	95	↓ At week 12→↑ response and OS	(Simeone *et al*, 2014)
Smoking status	Nivolumab		88	Current/former smokers→↑ response	(Hellmann *et al*, 2014)
ECOG PS	Nivolumab	Melanoma	98	<1 at baseline→↑ OS	(Nakamura *et al*, 2016)
	Nivolumab	NSCLC	175	<2 at baseline→↑ OS	(Bagley *et al*, 2017)
Liver metastases	Nivolumab	NSCLC	175	Presence at baseline→↓ OS	(Bagley *et al*, 2017)
irAE	Ipilimumab	Melanoma	139	Early irAE→↑ response	(Downey *et al*, 2007)
	Ipilimumab	Melanoma	298	No association with OS	(Horvat *et al*, 2015)
	Nivolumab	Melanoma	576	Any-grade AE→↑ response	(Weber *et al*, 2017)
	Nivolumab	Melanoma	148	Rash, vitiligo and any grade AE→↑ OS	(Freeman-Keller *et al*, 2016)
	Pembrolizumab	Melanoma	67	Vitiligo→↑ objective response	(Hua *et al*, 2016)
	Immunotherapy	Melanoma	322	vitiligo-like depigmentation→↑OS	(Teulings *et al*, 2015)
Body composition	Ipilimumab	Melanoma	84	Baseline sarcopenia or low muscle attenuation→severe treatment-related toxicity	(Daly *et al*, 2017)

Abbreviations: ECOG PS=Eastern Cooperative Oncology Group Performance Status; ICI=immune checkpoint inhibitor, irAE=immune-related adverse events; NSCLC=non-small-cell lung cancer; OS=overall survival; PFS=progression-free survival. Derived neutrophil/lymphocyte ratio=Absolute neutrophil count/(total leucocyte count–absolute neutrophil count).
